# Internet Use among Patients with Schizophrenia and Depression

**DOI:** 10.3390/ijerph19095695

**Published:** 2022-05-07

**Authors:** Nikola Žaja, Jakša Vukojević, Tvrtko Žarko, Marko Marelić, Domagoj Vidović, Tea Vukušić Rukavina

**Affiliations:** 1 University Psychiatric Hospital Vrapče, Bolnička cesta 32, 10000 Zagreb, Croatia; nikola.zaja@bolnica-vrapce.hr (N.Ž.); jaksa.vukojevic@bolnica-vrapce.hr (J.V.); tvrtko.zarko@bolnica-vrapce.hr (T.Ž.); domagoj.vidovic@bolnica-vrapce.hr (D.V.); 2 Andrija Štampar School of Public Health, School of Medicine, University of Zagreb, Rockefellerova ulica 4, 10000 Zagreb, Croatia; marko.marelic@snz.hr

**Keywords:** mental health, internet, digital health, internet-based intervention, psychiatry, mood disorders, psychotic disorders, cyber mental health

## Abstract

Background: The high and increasing prevalence of internet use in the general population and the significant burden of depression and schizophrenia urge us to investigate the patterns of internet use among patients with these illnesses. The aim of this study is to assess internet use and mental health-related internet use among patients suffering from schizophrenia and depression. Methods: A total of 104 patients with psychosis and 105 patients with depression were surveyed to assess their internet use and mental health-related internet use. Results: The majority of participants were internet users (87.6%), with 66.7% of internet users with psychosis and 71.4% of internet users with depression using it as a source of information on mental health. Participants with psychosis significantly more attributed the internet and mental health internet forums as helpful in coping with their mental illness and were more interested in the utilization of online mental health services than participants with depression. Conclusions: General internet use in patients with schizophrenia and depression corresponds with the internet use of the general population; however, they use it more often as a source of health information than the general population. Mental health service providers should offer more online interventions and treatment programs to patients with psychosis and depression, as our study suggests there is an unmet need for online mental health services for such patients.

## 1. Introduction

The internet is a global phenomenon that has, over the years, made its way into most aspects of human lives. An estimation for March 2021 is that 65.6% of the world population was using the internet at that time, with the 2000–2021 growth of 1331.9% [1]. Populations of North America and Europe are especially prone to internet use, with usage percentages of 94.6 and 87.2, respectively [1].

The internet is used for various activities, the popular ones being sending and receiving e-mails, finding information, reading online news, participating in social networks and making phone or video calls [2]. A very common activity is accessing health information online, with 50–70% of internet users searching for a specific answer to a specific health-related question [3]. Apart from information on a specific disease or illness, commonly accessed health information includes advice on diet, nutrition and fitness, and information on drugs, experimental treatment, health insurance, a particular doctor or hospital [4,5].

Results regarding the frequency of internet use among psychiatric patients demonstrate an increase over the years. In a 2008 study, there were 64.7% [6], in a 2015 study 79.5% [7], in a 2017 study 78% [8] and in a 2020 study 98.3% [9] of the included psychiatric patients had used the internet.

The general internet use of psychiatric patients is related to online e-mail services, search engines, news, social media, job-related information, recreational information, internet forums, meeting others, shopping, accessing music or movies and other purposes [6,7]. Stigmatizing health conditions are often concealed by people who suffer from them. Because of the anonymity, informality and accessibility of the internet, people who suffer from stigmatizing conditions, especially from psychiatric illnesses, are more prone to utilizing the internet for health-related matters [10].

An important internet-use aspect of people with psychiatric disorders is the use of social media. This is especially important for young people, considering the outspread of social media use and the significant prevalence of mental disorders [11] in this age group. In the context of mental health, the benefits of social media use include the enablement of expression of thoughts and feelings and the reception of social support, while an important disadvantage is an association with psychological distress, anxiety and depression [12].

Schizophrenia and depression are among the most common psychiatric disorders that, by negatively affecting psychosocial functioning and the quality of life, have a profound impact on the individual and society [13,14]. Schizophrenic disorders are generally characterized by inappropriate or blunted affect and by distortions of thinking and perception. The most important and most common psychopathological phenomena in schizophrenia include hallucinatory voices commenting or discussing the patient in the third person, delusional perception and delusions of control, thought echo, thought insertion or withdrawal, thought broadcasting, influence or passivity and negative symptoms [15]. Depression is a disorder characterized by a lowering of mood, reduction in energy and decrease in activity. Typical symptoms also include reduced capacity for enjoyment, interest, and concentration, marked tiredness, disturbed sleep and appetite, and reduced self-esteem and self-confidence [15].

In online support groups, mental health conditions, especially depression, are associated with more emotional-support content compared to nonterminal physical conditions, for which more informational content is present [16]. Depressed people tend to express their needs on the internet implicitly, often disclosing personal problems and asking for help indirectly [17]. People with psychotic disorders also often search for mental health-related information online [8]. The way this information is accessed is similar to the one of the general population [18], and these similarities are present in various aspects of internet use, including the perceived advantages and disadvantages of the internet [19]. Persons with psychotic disorders are also prone to social networking, aiming at establishing new relationships, maintaining existing ones and receiving online peer support [20]. For people with severe mental illnesses, the main barriers to internet utilization are internet-use-related expenses, lack of skills and cognitive difficulties [21].

The high and increasing prevalence of internet use [22,23], and the significant burden of depression and schizophrenia [24] in the population of Croatia require an investigation of the patterns of internet use among patients with these illnesses. The aim of this study is to assess internet use and mental-health-related internet use among patients suffering from schizophrenia and depression in Croatia.

## 2. Materials and Methods

### 2.1. Study Population

The research was conducted at the University Psychiatric Hospital Vrapče, Zagreb, Croatia, between December 2018 and February 2020. Inclusion criteria were: inpatient status at any of the hospital’s departments, informed consent to participate in the study, older than 18 years, leading clinical diagnosis of depression (F32 and F33) or schizophrenia, schizotypal, delusional, and other non-mood psychotic disorders (F20–F29), according to ICD-10 [15]. The exclusion criteria included insufficient knowledge of Croatian, illiteracy, acute inpatients (treated less than 10 days or treated at the psychiatric intensive care unit) and a cognitive impairment making the completion of the questionnaire impossible.

The study was approved by the Ethical review committee of the University Psychiatric Hospital Vrapče.

### 2.2. Questionnaire

We developed the questionnaire after conducting a literature review [6,7,8,9,25]. We then carefully reviewed questions used in previous studies and chose those that addressed the research question the most. Those questions were supplemented with additional internet-use-related questions. Socio-demographic questions were adapted from the 2003 Croatian Adult Health Survey [26]. Lastly, we revised and piloted the final draft of the questionnaire. Details on the type of questions included in the questionnaire are included in the Supplementary Materials.

The full questionnaire is available upon request from the corresponding author.

### 2.3. Procedure

Survey participants were approached at the departments where they were being treated. The invitation to take part in the study was distributed orally at patient group meetings. They were all provided with an informational sheet regarding the study, which also included information on the treatment and protection of the acquired data. Patients were given appropriate time to consider their agreement to participate and to ask questions, after which they signed informed consent or refused participation.

All questionnaires were completed in the presence of the study staff, which allowed providing explanations and instructions to the participants. Since participants were inpatients, they were instructed to assess their internet use and mental-health-related internet use prior to hospital admission. Before submitting the questionnaire, all participants were asked to check if they skipped answering any questions.

### 2.4. Data Analysis

The data were anonymized and treated in a confidential manner. Demographic data, as well as key variables, were summarized as descriptive statistics. Differences in categorical variables between groups were tested using chi-square or Fisher’s exact test as appropriate. Statistical significance of differences between groups in continuous variables was tested using *t*-test. Statistical significance of differences between groups in ordinal variables was tested using a Mann–Whitney U test. Multivariate regression analysis was performed to assess the influence of sociodemographic variables and illness type on the internet use and time spent online. Statistical analysis was performed in IBM SPSS statistics 25.

## 3. Results

### 3.1. Participation Rate

Of the 246 patients fulfilling the inclusion criteria who were approached, 33 refused participation and 4 were excluded because of their inability to complete the survey. The participation rate was 79.38% (104/131) in the schizophrenia, schizotypal, delusional, and other non-mood psychotic disorders group (SDG) and 91.3% (105/115) in the depression disorders group (DDG). The overall participation rate was 84.95% (209/246).

### 3.2. Sociodemographic Characteristics

In SDG, 51.9% of respondents were male and 48.1% female, with an average age of 32.47 years, while in DDG, 28.6% of respondents were male and 71.4% female, with an average age of 48.9 years. In both groups, most respondents completed high school and lived in urban areas, 64.4% and 76.9% in SDG and 64.8% and 82.9% in DDG, respectively. The majority of respondents in SDG were unmarried (74.0%), while the percentage of married or cohabiting persons in DDG was 44.8%. The sociodemographic characteristics of the sample are shown in Table 1. Significant differences among groups were determined for age (*p* = 0.000) and gender (*p* < 0.01). Differences in age and gender are shown in Table 2 and Table 3.

The majority of respondents had previously received inpatient psychiatric treatment (71.8%), with significantly more previously treated persons in DDG (79.0%) than in SDG (64.4%) (*χ^2^* = 5516; *df* = 1; *p* = 0.019). Participants in DDG also had a statistically significantly higher average number of years of psychiatric treatment (8.99 years) than in SDG (3.8 years) (*t* = −5.245; *df* = 170.069; *p* = 0.000). The comparison of psychiatric history between SDG and DDG groups is shown in Table 4.

### 3.3. General Internet Use

The majority of participants stated they used the internet (87.6%), with significantly more users in SDG (95.2%) than in DDG (80.0%) (*χ^2^* = 11.071; *df* = 1; *p* < 0.001). Mobile phones were the most frequently used devices for internet access in both groups, with a significantly more frequent usage in SDG (82.7%) than in DDG (65.7%) (*χ^2^* = 7.860; *df* = 1; *p* < 0.005). There was no significant difference in the average daily internet use with 190.97 min in the SDG and 168.25 min in the DDG (*t* = 1.033; *df* = 177; *p* = 0.303). Participants in SDG used the internet statistically significantly more often for correspondence (57.7%) than participants in DDG (43.8%) (*χ^2^* = 4029; *df* = 1; *p* = 0.045), and also used it statistically significantly more often for learning (50.0% vs. 25.7%) (*χ^2^* = 13,017; *df* = 1; *p* = 0.000) and playing video games (23.1% vs. 12.4%) (*χ^2^* = 4103; *df* = 1; *p* = 0.043). Only a small percentage of respondents who used the internet did not use social networks (5.8% in SG and 13.3% in DG) (*χ^2^* = 3455; *df* = 1; *p* = 0.063). General internet use habits are shown in Table 5.

Multivariate regression analysis did not find any of the sociodemographic variables associated with internet use. Likewise, the type of illness (depression or schizophrenia) does not seem to independently affect the likelihood of internet use (Table 6).

Older age was significantly associated with a lower likelihood of spending more time online (defined as daily internet use of 150 min or more, which was the median in the sample) (*p* < 0.001). Additionally, living in a rural setting is associated with a more than three times lower odds ratio for a daily internet use of 150 min or longer (*p* = 0.013). Gender did not show to be a significant variable in this regard. Likewise, the type of illness (depression or schizophrenia) does not seem to independently affect the amount of time spent online. Multivariate regression analysis of time spent online is shown in Table 7.

### 3.4. Menta-Health-Related Internet Use

The majority of internet-using participants used it as a means for obtaining mental health information (68.9%), with similar percentages of users in SDG (66.7%) and DDG (71.4%) (*χ^2^* = 0.480; *df* = 1; *p* = 0.488). Information on illnesses were the most frequently sought for mental health information in both groups, with a statistically significantly higher frequency in SDG (69.2%) than in DDG (51.4%) (*χ^2^* = 6916; *df* = 1; *p* = 0.009). Participants in SDG were statistically significantly more likely to find internet useful or partially useful in dealing with mental illness (76.8%) than participants in DDG (46.4%) (*χ^2^* = 14,261; *df* = 2; *p* = 0.001), and were also statistically significantly more often interested in using professional online mental health services (70.2% vs. 53.3%) (*χ^2^* = 6.286; *df* = 1; *p* = 0.012). Mental-health-related internet use and preferences are shown in Table 8.

The majority of internet-using participants used online mental health forums (62.2%), with a slightly higher percentage of users in DDG (68.6%) than in SDG (55.8%), but the difference was not statistically significant (*χ^2^* = 3642); *df* = 1; *p* = 0.056). Participants in SDG statistically significantly more often found online mental health forums useful or partially useful in dealing with mental illness (94.0%) than respondents in DDG (77.4%) (*χ^2^* = 10,777; *df* = 2; *p* = 0.005). Mental health internet forums usage is shown in Table 9.

## 4. Discussion

### 4.1. General Internet Use

The percentage of patients using the internet in our study (87.6%) showed an increase compared to the one observed in a previous similar research (79.5% and 78%) [8,25]. However, those studies were undertaken 5 and 7 years before ours, which could explain the difference. A 2014 German study found that internet use among psychiatric patients corresponds to one of the general population as 79.5% of patients were internet users with an average use of 13 h a week, while 77.2% of the general population were internet users and spent an average of 19.7 h a week online [7]. Our research sample also had a higher proportion of internet users compared to the Croatian general population (82%) [23], which was expected due to the slightly higher average age of the general population (43.4 years) [27]. The average daily internet use of 190.97 min in the SDG and 168.25 min in DDG is also comparable to the German study; however, data on time spent online in the Croatian general population were unavailable to the authors. A more recent German study showed 98.3% of psychiatric patients use the internet, with an average weekly use of 20.6 h [9].

Multivariate regression analysis revealed the influence of some of the sociodemographic variables on time spent online, with older age and living in a rural setting associated with a lower likelihood of spending more time online. However, we did not find any of the sociodemographic variables associated with the likelihood of being an internet user. A study on older adults in the United States of America found that minority status, combined with the lowest levels of socioeconomic status (SES), substantially reduced the odds of using the internet for health information and proved to be a source of the digital divide [28]. Previous studies also found that low and medium SES was associated with an increased prevalence of depression [29,30]. However, the odds of a new episode were lower than the odds of persisting depression [30]. Research on first-episode psychosis treatment showed better effectiveness of coordinated specialty care was associated with high SES [31]. Systematic reviews associated lower SES with worse sleep parameters (lower total sleep time, longer sleep latency, greater sleep fragmentation and higher variability in sleep onset and sleep latency) [32] and increased prevalence of obstructive sleep apnea [33]. As SES is an important factor with many health effects [34], and its implications on epidemiology and treatment outcomes of mental health disorders should be kept in mind by mental health service providers.

Although the majority of participants in both groups were social media users, the percentage of users was much lower than in the Croatian general population (73%) [23], but similar to the one found in previous research (47.8%) [25]. Facebook and YouTube were the most frequently used social media platforms in our study in both groups, with YouTube being predominant in SDG and Facebook in DDG. This is in contrast to a systematic review on internet use for social interaction by people with psychosis that found Facebook to be the most commonly accessed platform and YouTube as significantly less likely to be used by patients with psychosis [35].

### 4.2. Mental-Health-Related Use

The majority of internet-using participants in our study utilized the internet for mental health purposes, accessing the information on mental disorders and medication, searching for mental health services and accessing platforms with other patients and mental health professionals, with proportions similar to previous research [6,25,36].

According to Eurostat, 60% of internet users in Croatia sought health information online [37]. However, in our sample, 68.9% of all internet users used it to seek mental health information online, with 66.7 in SDG and 71.4% in DDG.

A German study found even higher percentages of psychiatric patients using the internet for mental health-related purposes [9]. They also concluded that the level of functioning was also much lower in patients who sought information on mental disorders and professional help from psychiatrists online than in those who did not use it in such manner [9].

A recent study comparing internet use in patients with depression and schizophrenia found similar proportions of internet use for mental-health-related issues as in our study (67.31% and 63.16%) [38]. They also found extended exposure to internet surfing for health-related information and the attribution of greater relevance to information found online as unfavorable to treatment adherence among depressive patients, while the opposite was observed among patients suffering from schizophrenia [38]. In our study, participants from both groups showed the highest interest in information on mental health disorders, which proved to be significantly higher in the SDG. Additionally, we observed a statistically significant tendency in SDG for the attribution of the internet as helpful in coping with their mental illness. A study on internet use in patients with psychosis found an association between higher levels of loneliness and the internet use for mental health information, a finding plausibly explained by online interaction as compensation for social isolation [39].

Previous findings on the internet’s role in coping with mental disorders were ambivalent, with lack of personal contact and low quality of online information as the most common reason for patients’ disregard of the internet’s helpfulness [25]. As the quality of health- and mental-health-related information on the internet is often poor [40,41], it is becoming increasingly problematic that only about 25% of internet health information seekers regularly and thoroughly check the accuracy of the obtained information [42]. Khazaal et al. reported that psychiatric patients were relatively confident about the quality of content on visited websites, but not aware of the quality criteria for such websites [6]. Moreover, the high cognitive demand for internet use is a serious barrier for its use by people with mental disorders since cognitive functioning is commonly impaired in this group [43].

The median daily time spent online observed in our study (150 min) raises questions about the negative consequences of extensive internet use. Many potentially harmful effects of frequent digital technology use include heightened attention-deficit symptoms, impaired emotional and social intelligence, technology addiction, social isolation, impaired brain development and disrupted sleep [44]. A Korean study showed that 22% of patients with schizophrenia spectrum disorders met the criteria for problematic internet use and that such persons were significantly more likely to have high levels of perceived stress and dysfunctional coping strategies [45]. High rates of psychiatric comorbidity, particularly behavioral, anxiety and mood disorders were also found in young subjects with internet addiction [46]. An association between problematic internet use and anxiety traits and anxiety state was found in a study on Tunisian students [47], while a study on Vietnamese adolescents revealed that excessive internet use (>4 h/day) was significantly associated with poor sleep quality, but not with depression or anxiety [48]. Children are also affected by greater electronic media use, which is associated with depressive symptoms [49]. These findings suggest clinicians should be aware of the adverse effects of extensive internet use and the intricate connections between mental disorders and internet use, as there is a possibility that some mental disorders might result from internet use and vice versa.

When considering their elevated risk for comorbid medical conditions, premature mortality and lower quality of general medical care [50,51], psychiatric patients’ health-related internet use probably puts them at even higher risk than other groups of people. As the results of our study implicate a significant trust in information obtained on mental health forums, the situation seems even more worrying.

### 4.3. Comparison of Internet Use between Patients with Schizophrenia and Depression

The groups of participants in our study differed based on their sociodemographic characteristics and the main one being age. SDG participants were significantly younger than DDG, which was a consequence of purposive convenience sampling. This finding mostly reflects the usual clinical course of these two groups of disorders, with the schizophrenia group of disorders having an onset of illness and peak of hospitalization rates earlier than depression disorders [13,14]. Furthermore, patients with depression were also statistically more likely to be previously psychiatrically treated and had more years of psychiatric treatment than patients with psychosis. The age difference is probably the main reason for the difference in the percentage of internet users too. The difference was statistically insignificant, similar to previous research that also found no difference in general internet use [25,38]. There was also a significant difference regarding gender between the two groups, but multivariate regression showed this did not affect the likelihood of internet use or the time spent online.

Questions on mental health internet use showed significant differences between the two groups. SDG participants more often regarded the internet and mental health internet forums as helpful in coping with their illness and were also more interested in finding information on mental illnesses online. Furthermore, they were more interested in the utilization of online mental health services than DDG participants. These findings are in line with previous research that found people with psychosis highly interested in obtaining mental health information online, with general websites, social networks and video streaming sites as important information sources [39]. Other studies found benefits of internet use for persons with psychosis, such as being a source of social contact and peer support, as well as having favorable impact on treatment adherence [35,38].

### 4.4. Limitations

This study included a sample of psychiatric patients receiving care in a university psychiatric hospital in an urban area and, therefore, does not necessarily represent patients in primary care or without any treatment, nor does it necessarily represent patients from rural areas. Including only patients older than 18 years is also one of the limitations of this study as there is a proportion of underage patients with schizophrenia and depression who might have different internet use preferences. The participants of this study were only inpatients, who usually have a more severe mental illness, which could impact the frequency and type of internet use. The questionnaire used in this study was not validated. It analyzes the internet use reported by patients and did not measure actual internet usage. The study took place before the COVID-19 pandemic, during which an increase in telepsychiatric services was observed, which could impact some of the findings.

## 5. Conclusions

General internet use in patients with schizophrenia and depression corresponds with the internet use of the general population. Social media are used by the majority of patients in both groups, but on a lower scale than the general population. Most of the internet-using patients in both groups have used the internet as a source of mental health information, which is a higher percentage than health-related internet use in the general population. A higher interest in information on mental illnesses was observed among patients suffering from schizophrenia. They also more often regarded the internet and mental health internet forums as helpful to cope with their illness and were also more interested in the utilization of online mental health services.

The tendency to rely on the internet in coping with mental illness could be a result of the low availability of mental health services. Although the majority of them have not previously used professional online mental health services, participants in both groups expressed a high interest in the utilization of such services. Since the study was undertaken before the COVID-19 pandemic, which saw an increase in telepsychiatric services worldwide [52], such discrepancy might be mitigated in the future. We believe this might lead to online mental health services becoming the dominant modality of providing psychiatric care, reaching more people in need of treatment than ever before. Our predictions are also supported by recent studies showing interest of the youth population in using online mental health services [53] and by the satisfaction of the mental health providers with such services [54]. Online mental health services could include online video conference-based psychiatric consultations and psychotherapy sessions, as well as asynchronous modalities, such as smartphone applications and therapist’s guidance via email, or a combination of different modalities [55]. Since mobile phones were the dominant access point to the internet in our study, this could grant healthcare providers the opportunity to offer mobile phone interventions and specialized applications, further expanding the reach. This could be even more relevant for patients with psychosis as their interest in online interventions was higher, as well as their tendency to rely on the internet in coping with their illness. The results from our study could be used as a basis for the development of specialized e-interventions for patients with psychosis and depression. 

## Figures and Tables

**Table 1:** Sociodemographic characteristics of the sample.

	Total (*n* = 209)	Schizophrenia Group (*n* = 104)	Depression Group (*n* = 105)
**Age**			
Mean (SD)	40.72 (14.635)	32.47 (11.693)	48.90 (12.548)
Median	39	30.00	51.00
Minimum	18	18	18
Maximum	72	72	72
**Gender**			
Male	84 (40.2%)	54 (51.9%)	30 (28.6%)
Female	125 (59.8%)	50 (48.1%)	75 (71.4%)
**Area of living**			
Urban	167 (79.9%)	80 (76.9%)	87 (82.9%)
Rural	41 (19.6%)	23 (22.1%)	18 (17.1%)
Abroad	1 (0.5%)	1 (1.0%)	0 (0%)
**Marital status**			
Married/Cohabiting	65 (31.1%)	18 (17.3%)	47 (44.8%)
Single	107 (51.2%)	77 (74.0%)	30 (28.6%)
Divorced	29 (13.9%)	5 (4.8%)	24 (22.9%)
Widowed	8 (3.8%)	4 (3.8%)	4 (3.8%)
**Educational degree**			
No school degree	1 (0.5%)	1 (1.0%)	0 (0.0%)
Elementary school	21 (10.0%)	11 (10.6%)	10 (9.5%)
High school	135 (64.6%)	67 (64.4%)	68 (64.8%)
College degree	13 (6.2%)	6 (5.8%)	7 (6.7%)
University or higher degree	38 (18.2%)	19 (18.3%)	19 (18.1%)
Unknown	1 (0.5%)	0 (0.0%)	1 (1.0%)
**Occupational status**			
Unemployed	70 (33.5%)	46 (44.2%)	24 (22.9%)
Housewife/-husband	10 (10%)	3 (2.9%)	7 (6.7%)
Employee	67 (32.1%)	27 (26.0%)	40 (38.1%)
Self-employed	3 (1.4%)	3 (2.9%)	0 (0.0%)
Manager, director, principal	4 (1.9%)	3 (2.9%)	1 (1.0%)
Military or police force member	1 (0.5%)	0 (0.0%)	1 (1.0%)
Retiree	34 (16.3%)	9 (8.7%)	25 (23.8%)
Student	17 (8.1%)	12 (11.5%)	5 (4.8%)
Other	3 (1.4%)	1 (1.0%)	2 (1.9%)

SD = standard deviation.

**Table 2:** The difference in age between the two groups.

	$\bar{x}$	*s*	*df*	*t*	*p*
Schizophrenia group	32.47	11.693	207	−9.787	0.000 ***
Depression group	48.90	12.548			

*** *p* < 0.001.

**Table 3:** The difference in gender between the two groups.

	Schizophrenia Group *n* (%)	Depression Group *n* (%)	*χ^2^*	*df*	*p*
Male	54 (51.9)	30 (28.6)	11.853	1	0.001 **
Female	50 (48.1)	75 (71.4)			

** *p* < 0.01.

**Table 4:** Psychiatric history of the sample.

	Total (*n* = 209)	Schizophrenia Group (*n* = 104)	Depression Group (*n* = 105)	Statistical Test
Previously psychiatrically treated				^a^ *χ*^2^; *df*; *p*
Yes	150 (71.8%)	67 (64.4%)	83 (79.0%)	5.516; 1; 0.019 *
No	59 (28.2%)	37 (35.6%)	22 (21.0%)	
Number of previous hospital psychiatric treatments				^b^*t*; *df*; *p*
Mean (SD)	5.28 (8.180)	4.04 (5.917)	6.28 (9.547)	−1.672; 148; 0.097
Median	3	2.00	4.00	
Minimum	1	1	1	
Maximum	80	33	80	
How many years under psychiatric treatment				^b^*t*; *df*; *p*
Mean (SD)	6.40 (7.609)	3.80 (5.190)	8.99 (8.694)	−5.245; 170.069; 0.000 ***
Median	4	1.75	6	
Minimum	0.08	0.08	0.08	
Maximum	50	23	50	
In psychiatric treatment				^c^*U*; *p*
Less than a year	53 (25.4%)	40 (38.5%)	13 (12.4%)	3310.00; 0.000 ***
1–3 years	38 (18.2%)	22 (21.2%)	16 (15.2%)	
3–5 years	37 (17.7%)	17 (16.3%)	20 (19.0%)	
5 years and more	81 (38.7%)	25 (24.0%)	56 (53.3%)	

* *p* < 0.05; *** *p* < 0.001, SD = standard deviation, ^a^ chi-square test, ^b^ *t*-test, ^c^ Mann–Whitney *U* test.

**Table 5:** General internet use habits.

	Total	Schizophrenia Group *n* (%)	Depression Group *n* (%)	*χ*^2^; *df*; *p*
Purpose of internet use				
Social media	112 (53.6)	58 (55.8)	54 (51.4)	0.396; 1; 0.529
Messaging	106 (50.7)	60 (57.7)	46 (43.8)	4.029; 1; 0.045 *
Reading news	101 (48.3)	56 (53.8)	45 (42.9)	2.527; 1; 0.112
Studying	79 (37.8)	52 (50.0)	27 (25.7)	13.017; 1; 0.000 ***
Videogames	37 (17.7)	24 (23.1)	13 (12.4)	4.103; 1; 0.043 *
Other	14 (6.7)	9 (8.7)	5 (4.8)	1.266; 1; 0.260
Daily used social media				
None	20 (9.6)	6 (5.8)	14 (13.3)	3.455; 1; 0.063
Facebook	124 (59.3)	64 (61.5)	60 (57.1)	0.418; 1; 0.518
Twitter	2 (1.0)	2 (1.9)	0 (0)	0.246 ^a^
Instagram	43 (20.6)	29 (27.9)	14 (13.3)	6.770; 1; 0.009 **
Snapchat	10 (4.8)	3 (2.9)	7 (6.7)	0.332 ^a^
YouTube	116 (55.5)	75 (72.1)	41 (39.0)	23.133; 1; 0.000 ***
Other	3 (1.4)	1 (1.0)	2 (1.9)	1.000 ^a^
Time spent on social media daily				
Less than 1 h	72 (39.8)	37 (38.1)	35 (41.7)	
1–3 h	87 (48.1)	45 (46.4)	42 (50.0)	2.145; 2; 0.342
More than 3 h	22 (12.2)	15 (15.5)	7 (8.3)	

* *p* < 0.05; ** *p* < 0.01; *** *p* < 0.001; ^a^ Fisher’s exact test.

**Table 6:** Internet use (yes or no)—multivariate regression analysis.

Variable	Odds Ratio	95% CI	*p*-Value
Age	1.025	0.892–1.179	0.726
Sex	0.369	0.031–4.361	0.429
Rural living setting	5.749	0.452–73.189	0.178
Marital status	1.143	0.260–5.032	0.860
Educational status	0.235	0.032–1.717	0.154
Occupational status	4.095	0.698–23.016	0.118
Illness type (schizophrenia or depression)	3.021	0.080–113.5	0.550

CI = confidence interval.

**Table 7:** Time spent online (use of internet 150 min or more per day)—multivariate regression analysis.

Variables	Odds Ratio	95% CI	*p*-Value
Age	0.955	0.931–0.980	<0.001 ***
Sex	0.870	0.453–1.669	0.675
Rural living setting	0.305	0.120–0.775	0.013 *
Marital status	1.563	0.950–2.570	0.079
Educational status	0.801	0.462–1.391	0.431
Occupational status	0.784	0.552–1.113	0.173
Illness type (schizophrenia or depression)	0.840	0.384–1.839	0.663

* p < 0.05; *** *p* < 0.001; CI = confidence interval.

**Table 8:** Mental-health-related internet use and preferences.

	Total	Schizophrenia Group *n* (%)	Depression Group *n* (%)	*χ*^2^; *df*; *p*
Internet as a source of information on mental health				
Yes	126 (68.9)	66 (66.7)	60 (71.4)	0.480; 1; 0.488
No	57 (31.1)	33 (33.3)	24 (28.6)	
Type of mental health information interested in on the internet				
Information on illnesses	126 (60.3)	72 (69.2)	54 (51.4)	6.916; 1; 0.009 **
Information on medication	96 (45.9)	49 (47.1)	47 (44.8)	0.117; 1; 0.733
Information on treatment and psychiatric institutions	57 (27.3)	30 (28.6)	27 (25.7)	0.258; 1; 0.611
Communication with others with mental disorders	47 (22.5)	29 (27.9)	18 (17.1)	3.459; 1; 0.063
Online health services	36 (17.2)	18 (17.3)	18 (17.1)	0.001; 1; 0.975
Other	6 (2.9)	2 (1.9)	4 (3.8)	0.683 ^a^
Internet helpful in coping with the mental disorder				
Yes	51 (27.9)	39 (39.4)	12 (14.3)	14.261; 2; 0.001 **
Partially	82 (44.8)	37 (37.4)	27 (32.1)	
No	50 (27.3)	23 (23.2)	45 (53.6)	
Interested in professional online mental health service				
Yes	129 (61.7)	73 (70.2)	56 (53.3)	6.286; 1; 0.012 *
No	80 (38.3)	31 (29.8)	49 (46.7)	
Ever used professional online health services				
Yes	13 (7.1)	8 (8.1)	5 (6.0)	0.288; 1; 0.592
No	169 (92.9)	91 (91.9)	78 (94.0)	

* *p* < 0.05; ** *p* < 0.01; ^a^ Fisher’s exact test.

**Table 9:** Mental health internet forums usage.

	Total	Schizophrenia Group *n* (%)	Depression Group *n* (%)	*χ*^2^; *df*; *p*
Reasons for using mental health internet forums				
Does not use	79 (37.8)	46 (44.2)	33 (31.4)	3.642; 1; 0.056
Sharing useful information	26 (12.4)	16 (15.4)	10 (9.5)	1.648; 1; 0.199
Receiving useful information	89 (42.6)	49 (47.1)	40 (38.1)	1.739; 1; 0.187
Providing emotional support	16 (7.7)	8 (7.7)	8 (7.6)	0.000; 1; 0.984
Receiving emotional support	19 (9.1)	13 (12.5)	6 (5.7)	2.911; 1; 0.088
Other	3 (1.4)	0 (0.0)	3 (2.9)	0.249 ^a^
Mental health internet forums help cope with the mental illness				
Yes	42 (23.0)	26 (26.3)	16 (19.0)	10.777; 2; 0.005 **
Partially	116 (63.4)	67 (67.7)	49 (58.3)	
No	25 (13.7)	6 (6.1)	19 (22.6)	
Finding information on mental health internet forums accurate				
Yes	78 (42.6)	43 (43.4)	35 (41.7)	0.058; 1; 0.810
No	105 (57.4)	56 (56.6)	49 (58.3)	

** *p* < 0.01; ^a^ Fisher’s Exact test.

## Data Availability

Data are available upon reasonable request from the corresponding author.

## Supplementary Materials

The following supporting information can be downloaded at: https://www.mdpi.com/article/10.3390/ijerph19095695/s1. Types of questions in the study questionnaire.
