# Supplementary material for: Internet Use among Patients with Schizophrenia and Depression
Source: Int J Environ Res Public Health. 2022 May 7;19(9):5695. doi: 10.3390/ijerph19095695 (PMC9104824; doi:10.3390/ijerph19095695)
Supplement: Supplementary file 1 [file ijerph-19-05695-s001.zip › ijerph-1650909-supplementary.pdf]

Types of questions in the study questionnaire

Our study questionnaire consists of 37 items divided into three sections: general internet use, mental health-related internet use and socio-demographic characteristics (with a history of previous hospitalizations in psychiatric institutions). Patients who answered that they do not use the internet answered only socio-demographic characteristics questions and three mental health-related internet use questions. In order to facilitate completion of the survey for internet non-users, those three questions from the mental health-related internet use section were inserted at the start of the survey, followed by the first general internet use question, assessing the internet use.

Internet use-related questions included 11 questions (one yes-or-on question, five multiple-choice questions and five time-specifications) and assessed general internet use, means for accessing the internet, usage of the mobile phones, utilization of social networks and playing videogames.

Mental health-related internet use was examined by 17 questions (two open-ended questions, one Likert scale question, nine multiple-choice questions and five yes-or-no questions), assessing peer communication, and seeking mental health-related information and services online.

Socio-demographic characteristics were collected by 9 questions (seven multiple-choice questions, one frequency-specification and one time-specifications), assessing age, gender, place of living, marital status, education level, and occupation, but also previous psychiatric treatment.
